# Antioxidant, Anti-Inflammation, and Melanogenesis Inhibition of Sang 5 CMU Rice (*Oryza sativa*) Byproduct for Cosmetic Applications

**DOI:** 10.3390/plants13131795

**Published:** 2024-06-28

**Authors:** Pichchapa Linsaenkart, Warintorn Ruksiriwanich, Anurak Muangsanguan, Sarana Rose Sommano, Korawan Sringarm, Chaiwat Arjin, Pornchai Rachtanapun, Kittisak Jantanasakulwong, Juan M. Castagnini, Romchat Chutoprapat, Korawinwich Boonpisuttinant

**Affiliations:** 1 Department of Pharmaceutical Sciences, Faculty of Pharmacy, Chiang Mai University, Chiang Mai 50200, Thailand; pichchapa_li@cmu.ac.th (P.L.); anurak_m@cmu.ac.th (A.M.); 2 Cluster of Valorization and Bio-Green Transformation for Translation Research Innovation of Raw Materials and Products, Chiang Mai University, Chiang Mai 50200, Thailand; sarana.s@cmu.ac.th (S.R.S.); korawan.s@cmu.ac.th (K.S.); 3 Center of Excellence in Agro Bio-Circular-Green Industry (Agro BCG), Chiang Mai University, Chiang Mai 50200, Thailand; pornchai.r@cmu.ac.th (P.R.); kittisak.jan@cmu.ac.th (K.J.); 4 Department of Plant and Soil Sciences, Faculty of Agriculture, Chiang Mai University, Chiang Mai 50200, Thailand; 5 Department of Animal and Aquatic Sciences, Faculty of Agriculture, Chiang Mai University, Chiang Mai 50200, Thailand; 6 School of Agro-Industry, Faculty of Agro-Industry, Chiang Mai University, Chiang Mai 50100, Thailand; 7 Research Group in Innovative Technologies for Sustainable Food (ALISOST), Department of Preventive Medicine and Public Health, Food Science, Toxicology and Forensic Medicine, Faculty of Pharmacy, Universitat de València, Avenida Vicent Andrés Estellés s/n, 46100 Burjassot, Spain; juan.castagnini@uv.es; 8 Department of Pharmaceutics and Industrial Pharmacy, Faculty of Pharmaceutical Sciences, Chulalongkorn University, Bangkok 10300, Thailand; romchat.c@pharm.chula.ac.th; 9 Innovative Natural Products from Thai Wisdoms (INPTW), Faculty of Integrative Medicine, Rajamangala University of Technology Thanyaburi, Pathum Thani 12130, Thailand; korawinwich_b@rmutt.ac.th

**Keywords:** collagen production, hyperpigmentation, *IL-6*, MMP-2, *NRF2*, oxidative stress, rice bran, rice husk, *TYR*

## Abstract

Prolonged exposure to environmental oxidative stress can result in visible signs of skin aging such as wrinkles, hyperpigmentation, and thinning of the skin. *Oryza sativa* variety Sang 5 CMU, an inbred rice cultivar from northern Thailand, contains phenolic and flavonoid compounds in its bran and husk portions that are known for their natural antioxidant properties. In this study, we evaluated the cosmetic properties of crude extracts from rice bran and husk of Sang 5 CMU, focusing on antioxidant, anti-inflammatory, anti-melanogenesis, and collagen-regulating properties. Our findings suggest that both extracts possess antioxidant potential against DPPH, ABTS radicals, and metal ions. Additionally, they could downregulate TBARS levels from 125% to 100% of the control, approximately, while increasing the expression of genes related to the NRF2-mediated antioxidant pathway, such as *NRF2* and *HO-1*, in H_2_O_2_-induced human fibroblast cells. Notably, rice bran and husk extracts could increase mRNA levels of *HO-1* more greatly than the standard L-ascorbic acid, by about 1.29 and 1.07 times, respectively. Furthermore, the crude extracts exhibited anti-inflammatory activity by suppressing nitric oxide production in both mouse macrophage and human fibroblast cells. Specifically, the bran and husk extracts inhibited the gene expression of the inflammatory cytokine *IL-6* in LPS-induced inflammation in fibroblasts. Moreover, both extracts demonstrated potential for inhibiting melanin production and intracellular tyrosinase activity in human melanoma cells by decreasing the expression of the transcription factor *MITF* and the pigmentary genes *TYR*, *TRP-1*, and *DCT*. They also exhibit collagen-stimulating effects by reducing MMP-2 expression in H_2_O_2_-induced fibroblasts from 135% to 80% of the control, approximately, and increasing the gene associated with type I collagen production, *COL1A1*. Overall, the rice bran and husk extracts of Sang 5 CMU showed promise as effective natural ingredients for cosmetic applications.

## 1. Introduction

The skin serves as the first barrier protecting the human body from the environment. Antioxidant pathways play a crucial role as the primary defense mechanisms against external stimuli. Prolonged exposure to oxidative stress may result in damaged cells and disrupt the homeostasis of skin function. Visible signs of skin aging, including wrinkles, melasma, and skin thinning, can manifest as a consequence [1,2].

Typically, the nuclear factor erythroid 2-related factor 2 (NRF2)-mediated pathway is one of the main defense mechanisms for skin damage. Activation of this pathway leads to the nuclear translocation of NRF2, resulting in the expression of antioxidant proteins including heme oxygenase-1 (HO-1) and NAD(P)H dehydrogenase quinone 1 (NQO-1) [3]. Consequently, the expression of antioxidant enzymes such as HO-1 represents the second phase of antioxidant defenses [4].

Skin inflammation often occurs due to oxidative damage, allergic reactions, or pathogen invasion. Various reactive oxygen species (ROS), reactive nitrogen species (RNS), and inflammatory cytokines such as interleukin (IL)-1β and IL-6 are produced by skin cells and attract the innate immune cells to the site of skin infection [5]. Moreover, skin inflammation after exposure to UV light or environmental stressors is associated with NRF2 depletion. Enhancing these antioxidant mechanisms is an important goal for preventing skin aging as well as skin-related diseases [4].

Melanin pigments produced in melanocytes are part of the response to oxidative stress or inflammatory cytokine exposure such as non-radical ROS, endogenous hydrogen peroxide (H_2_O_2_), or nitric oxide (NO) products [6]. Prolonged and unprotected exposure to external factors might lead to chronic dermatological issues such as melasma [1]. In particular, the downregulation of antioxidant pathways like NRF2 and HO-1 levels is associated with skin hyperpigmentation [7]. Melanocyte-inducing transcription factor (*MITF*) is known as a key regulator of melanogenesis in controlling the expression of pigmentary genes such as tyrosinase (*TYR*), tyrosinase-related protein-1 (*TRP-1*), and tyrosinase-related protein-2, also known as dopachrome tautomerase (*TRP-2*/*DCT*), respectively [8].

Skin aging is related to the destruction of the dermal extracellular matrix (ECM). The major components in ECM structure are collagen and elastin, which provide skin with tensile strength and elasticity, respectively. Exposure to oxidative stress can induce senescence in normal human dermal fibroblast cells, leading to the secretion of matrix metalloproteinases (MMPs) that degrade collagen and elastin. Type I collagen (COL1), which constitutes nearly 90% of adult skin, is particularly affected [2,9]. Previous reports have shown that senescence skin cells exhibited overexpression of MMPs and a decrease in collagen synthesis, resulting in alterations to the ECM-supporting structure [10].

Furthermore, there are a large number of natural antioxidants derived from plant resources that have been utilized for skin protection. Phenolic and flavonoid compounds are considered to possess effective antioxidant capabilities and fewer side effects, such as skin itching or erythema, compared to anti-aging chemical compounds such as retinoids [4,11].

Rice (*Oryza sativa*) is one of the most widely produced food crops in the world. The forecast for global paddy rice production suggests an increase from 55 million tons in 2022 to 577 million tons by 2032 [12]. Rice by-product has been widely used in cosmetic applications for the benefits from its anti-wrinkle [13] or anti-hair loss [14] properties. Rice husk, a major byproduct constituting roughly 20% of rice weight, is initially removed from paddy rice during the milling process. Afterward, the rice bran is removed to obtain white rice. In addition, rice bran oil is further extracted from rice bran, serving purposes in cooking and as a health supplement. During this process, the defatted rice bran is typically generated as waste [15,16]. Thus, adding value to agricultural byproducts such as defatted rice bran and husk will have a positive impact on the environment and reduce the costs associated with waste removal.

Sang 5 CMU is one of the inbred rice varieties in the northern Thai region. It was registered under legislative protection in November 2020. Our previous projects focused on the analysis of bioactive compounds in rice bran and husk among the 12 inbred rice varieties. We used ethanol, one of the GRAS (generally recognized as safe) solvents, for the extraction of bioactive compounds from the samples. While water is the safest and cheapest solvent, ethanol is more suitable for the extraction of poorly soluble bioactive compounds, such as polyphenols, from plant-based samples [17]. We found that the defatted rice bran of Sang 5 CMU is primarily composed of quercetin, chlorogenic acid, naringin, or epicatechin. Furthermore, in the rice husk part of Sang 5 CMU, epigallocatechin gallate is notably predominant, as it is among all the 12 northern rice cultivars [18,19]. These phenolic and flavonoid compounds have been recognized as natural antioxidant agents. Due to their natural phenolic structure, hydroxyl groups normally function as radical scavengers [20].

Regarding these findings, the ethanolic-defatted rice bran (DFRB) and husk (H) extracts of Sang 5 CMU might possess biological activities for addressing skin aesthetic problems. In this study, we evaluated the potential candidates for cosmetic applications by investigating their effects on various pathways. We first screened the antioxidant potential of Sang 5 CMU rice extracts through DPPH, ABTS, and iron chelating assays. We further examined their antioxidant potential by evaluating the upregulation of genes related to the NRF2-mediated pathway, including *NRF2* and *HO-1*. Furthermore, we investigated the anti-inflammatory activity by examining the suppression of nitric oxide production and genes related to pro-inflammatory cytokines, such as *IL-1β* and *IL-6*. Additionally, we analyzed their anti-melanogenesis activity by assessing intracellular melanin content, the tyrosinase activity assay, and the downregulation of genes associated with melanogenesis, including *MITF*, *TYR*, *TRP-1*, and *DCT*. Moreover, we evaluated their collagen-stimulating effects by observing the downregulation of MMP-2 using gelatin zymography and the upregulation of the gene related to type I collagen production, *COL1A1*.

## 2. Results

### 2.1. Crude Extracts Preparation

The crude extract of defatted rice bran appeared as a greasy, dark brown paste. In the case of rice husk, the sample exhibited a dark brown, sticky, and coarse paste consistency. The yields obtained from the maceration of defatted rice bran (DFRB) and rice husk (H) were 7.13% and 1.79% based on dry weight, respectively. According to our previous chemical composition analysis, the rice bran of Sang 5 CMU contains the following phenolic and flavonoid compounds [18,19], as shown in Table 1.

### 2.2. Scavenging Activity

#### 2.2.1. Antioxidant Effects of Sang 5 CMU Extracts against DPPH, ABTS Radicals, and Ferrous Ion

To screen the antioxidant effects of extracts, DPPH and ABTS radicals scavenging and iron chelating assays were conducted. The results are expressed as IC_50_ values. A high IC_50_ value is regarded as indicative of poor scavenging activity towards radicals or metal ions. As shown in Table 2, DFRB possessed stronger antioxidant activity than H via the DPPH assay (*p* < 0.05), while both DFRB and H showed no significant scavenging activity against the ABTS radical. However, H showed more effective iron chelation than DRFB (*p* < 0.05). However, higher concentrations of crude extracts are required to inhibit 50% of the activity of free radicals or metal ions to achieve the same level of inhibition as the standard scavenger, (+/−)-6-hydroxy-2,5,7,8-tetramethyl-chroman-2-carboxylic acid (Trolox), and the chelator, ethylene diamine tetra acetic acid (EDTA). To further determine the scavenging effect in human fibroblast cells, we measured MDA production after exposure to sources of oxidative stress, such as H_2_O_2_.

#### 2.2.2. Antioxidant Effects of Sang 5 CMU Extracts after H_2_O_2_-induced Oxidative Stress in Fibroblast Cells

**Figure 1:**
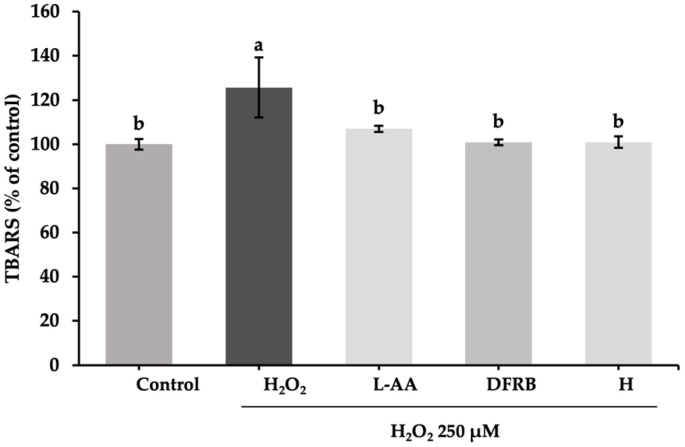
Inhibitory effects of defatted rice bran (DFRB) and husk (H) extracts of *Oryza sativa* cv. Sang 5 CMU on TBARS release from fibroblast cells after exposure to 250 μM hydrogen peroxide (H_2_O_2_). L-ascorbic acid (L-AA) was used as a positive control. Experiments were performed in triplicate. Bars represent mean ± standard deviation. Different superscript letters (a and b) were significantly different at *p* < 0.05, while the same letter showed no significant difference according to a one-way ANOVA test, followed by LSD post hoc test.

The highest concentration for further experiments was selected based on the SRB cytotoxic assay, ensuring non-cytotoxicity (>80% viability of fibroblast cells) at 1 mg/mL for each extract [14]. The measurement of TBARS levels, i.e., the adduction between MDA and TBA reagents, is a marker for the lipid peroxidation process in cells. After stimulation with H_2_O_2_, the levels of TBARS produced from cells significantly increased, up to 125.68 ± 13.51% of the control, compared to those of untreated cells (*p* < 0.05). As shown in Figure 1, DFRB and H extracts showed noticeably reduced TBARS production in H_2_O_2_-induced fibroblast cells (*p* < 0.05). Values were found to be 100.92 ± 1.30 and 100.92 ± 2.60% of the control, respectively. Additionally, the scavenging effects of both extracts were comparable to the L-ascorbic acid treatment group (107.03 ± 1.40% of control).

#### 2.2.3. Effects of Sang 5 CMU Extracts on Gene Expression of Antioxidant-Related Genes

**Figure 2:**
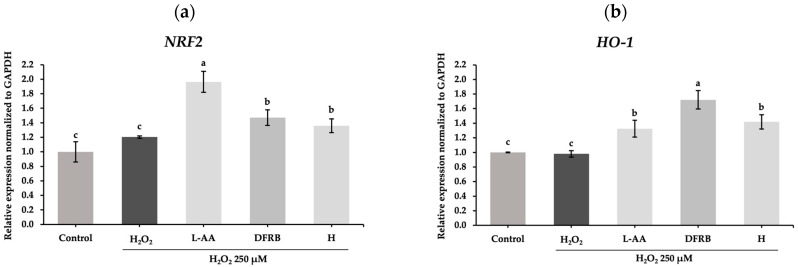
Effects of defatted rice bran (DFRB) and husk (H) extracts of *Oryza sativa* cv. Sang 5 CMU on the gene expression of antioxidant-related genes: (**a**) *NRF2*; (**b**) *HO-1* in human fibroblast cells after exposure to 250 μM hydrogen peroxide (H_2_O_2_). L-ascorbic acid (L-AA) was used as a positive control. Experiments were performed in triplicate. Bars represent mean ± standard deviation. Different superscript letters (a, b, and c) were significantly different at *p* < 0.05, while the same letter showed no significant difference according to a one-way ANOVA test, followed by LSD post hoc test.

To confirm the antioxidant effect, mRNA analysis of genes encoding *NRF2,* a transcription factor involved in cellular defense against oxidative stress, and *HO-1*, one of the antioxidant enzymes, was conducted. After cell exposure to a source of oxidative stress, H_2_O_2_, the relative expression of *HO-1* was slightly downregulated to 0.98 ± 0.04 compared to the untreated group. Consistent with the results from the TBARS assay, DFRB and H extracts influenced the expression of *NRF2* in H_2_O_2_-induced fibroblasts with relative expression values of 1.47 ± 0.11 and 1.36 ± 0.10, respectively, as illustrated in Figure 2a. These results indicated that DFRB and H treatments can increase mRNA levels of *NRF2* by approximately 0.75-fold and 0.69-fold compared to L-AA treatment, respectively. Additionally, the mRNA levels of *HO-1* considerably rose to 1.33 ± 0.12, 1.72 ± 0.13, and 1.42 ± 0.10, in the L-AA, DFRB, and H treatment groups, respectively, compared to the untreated and H_2_O_2_-treatment groups (Figure 2b). Therefore, DFRB and H treatments can increase mRNA levels of *HO-1* more greatly than the standard L-AA, by about 1.29 and 1.07 times, respectively.

### 2.3. Anti-Inflammation

#### 2.3.1. Anti-Inflammation Effects of Sang 5 CMU Extracts after LPS-Induced Inflammation in Murine Macrophage and Human Fibroblast Cells

**Figure 3:**
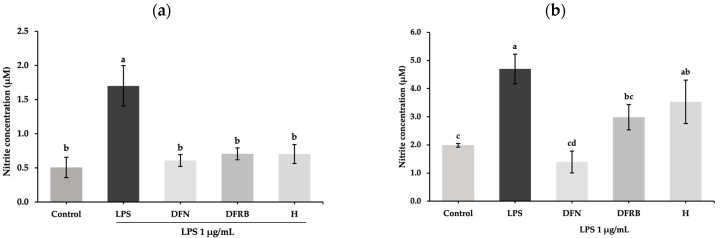
Inhibitory effects of defatted rice bran (DFRB) and husk (H) extracts of *Oryza sativa* cv. Sang 5 CMU on nitrite release from (**a**) murine RAW 264.7 macrophage and (**b**) human fibroblast cells after exposure to 1 μg/mL lipopolysaccharides (LPS). Diclofenac sodium (DFN) was used as a positive control. Experiments were performed in triplicate. Bars represent mean ± standard deviation. Different superscript letters (a, b, c, and d) were significantly different at *p* < 0.05, while the same letter showed no significant difference according to a one-way ANOVA test, followed by LSD post hoc test.

The non-toxic concentrations of Sang 5 CMU extracts were chosen through the SRB assay. After treating RAW 264.7 macrophage cells with extracts, concentrations of 0.03125, 0.0625, 0.125, 0.25, 0.5, and 1 mg/mL for DFRB and H extracts demonstrated cell viability above 80%. Consequently, the highest concentration at 1 mg/mL for each extract was selected for the further tests. The nitrite concentration assay in RAW 264.7 was first measured. The results showed that the levels of nitrite in the LPS treatment group (1.70 ± 0.30 μM) were notably higher than those in the untreated group (*p* < 0.05). As demonstrated in Figure 3a, DFRB and H extracts significantly suppressed the nitrite production in LPS-induced macrophage cells at 0.71 ± 0.01 and 0.70 ± 0.14 μM, respectively (*p* < 0.05). The anti-inflammatory effects of both extracts were comparable to those of the DFN treatment group (0.61 ± 0.01 μM). Apart from the effects of crude extracts in murine macrophage, we also performed the nitrite concentration assay in human fibroblast cells to confirm the application of extracts to the skin. As shown in Figure 3b, the levels of nitrite concentration in the LPS-treated group were considerably upregulated to 4.70 ± 0.53 μM compared to the untreated group (*p* < 0.05). In the DFRB pre-treatment group, nitrite production was suppressed to 2.99 ± 0.45 μM in LPS-induced fibroblasts (*p* < 0.05).

#### 2.3.2. Effects of Sang 5 CMU Extracts on Gene Expression of Inflammation-Related Genes

**Figure 4:**
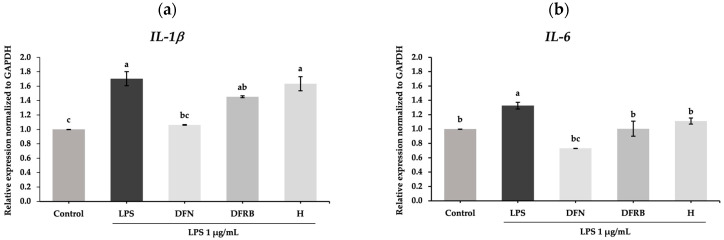
Effects of defatted rice bran (DFRB) and husk (H) extracts of *Oryza sativa* cv. Sang 5 CMU on the gene expression of inflammation-related genes (**a**) *IL-1β* and (**b**) *IL-6* in human fibroblast cells after exposure to 1 μg/mL lipopolysaccharides (LPS). Diclofenac sodium (DFN) was used as a positive control. Experiments were performed in triplicate. Bars represent mean ± standard deviation. Different superscript letters (a, b, and c) were significantly different at *p* < 0.05, while the same letter showed no significant difference according to a one-way ANOVA test, followed by LSD post hoc test.

Nitric oxide (NO) is a mediator produced during cutaneous inflammation. The expression levels of NO donors following inflammation within the epidermis and dermis layers could elevate the levels of inflammatory cytokines such as IL-1β and IL-6 [21]. Subsequently, the expression of genes related to inflammatory cytokines was further evaluated in LPS-induced human fibroblast cells. After LPS exposure to fibroblast cells, the mRNA levels of pro-inflammatory cytokines, including *IL-1β* and *IL-6*, were markedly increased to 1.70 ± 0.10 and 1.33 ± 0.05, respectively, compared to the control group (*p* < 0.05). As shown in Figure 4a, the results indicated that DFRB and H extracts slightly declined the expression of *IL-1β* to 1.45 ± 0.01 and 1.63 ± 0.10, respectively. Notably, concerning the mRNA expression of *IL-6*, DFRB and H extracts could significantly downregulate the levels of *IL-6* in LPS-stimulated fibroblasts, with relative expression values of 1.00 ± 0.11 and 1.11 ± 0.04, respectively, with a comparable level of DFN treatment (0.73 ± 0.00), as demonstrated in Figure 4b. DFRB and H treatments can decrease mRNA levels of *IL-6* by approximately 1.37 and 1.52 times compared to DFN treatment, respectively.

### 2.4. Anti-Melanogenesis Activity

#### 2.4.1. Inhibitory Effects of Sang 5 CMU Extracts against Mushroom Tyrosinase Enzyme

The mushroom tyrosinase inhibition assay is widely used as a screening test to investigate potential anti-melanogenesis agents. As shown in Table 3, the IC_50_ value of H extract was comparable to that of arbutin. However, we also examined the inhibition effects of both extracts in human melanoma cells.

#### 2.4.2. Inhibitory Effects of Sang 5 CMU Extracts after IBMX-Induced Melanogenesis in Human Melanoma Cells

**Figure 5:**
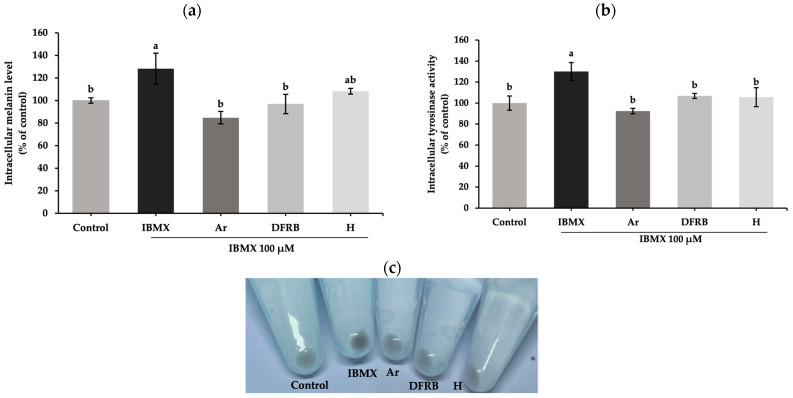
Inhibitory effects of defatted rice bran (DFRB) and husk (H) extracts of *Oryza sativa* cv. Sang 5 CMU on (**a**) intracellular melanin content and (**b**) tyrosinase activity in human melanoma cells after exposure to 100 μM 3-isobutyl-1-methylxanthine (IBMX). (**c**) Cell pellets after treatment with each sample for 48 h. Arbutin (Ar) was used as a positive control. Experiments were performed in triplicate. Bars represent mean ± standard deviation. Different superscript letters (a and b) were significantly different at *p* < 0.05, while the same letter showed no significant difference according to a one-way ANOVA test, followed by LSD post hoc test.

Before the test, the optimal concentration of each sample was determined based on the cell viability test. A concentration of 0.03125 mg/mL for both extracts resulted in the G-361 cell viability exceeding 80%. Consequently, the concentration of 0.03125 mg/mL for DFRB and H was chosen for subsequent tests. Regarding the melanogenesis activity, the measurement involved assessing intracellular melanin levels and tyrosinase activity in melanoma cells (Figure 5). IBMX was added to stimulate melanogenesis activity within the melanoma cells. The inhibition on melanogenesis activity was reported as relative percentages of the untreated group. The IBMX treatment group showed a considerable rise in melanin content and tyrosinase activity levels at 128.21 ± 13.72 and 130.07 ± 8.39% of the control, respectively. After pre-treatment with samples, DFRB and H substantially decreased the levels of melanin content (96.94 ± 8.60 and 108.32 ± 2.54% of control) (Figure 5a). As shown in Figure 5b, the intracellular tyrosinase activity in IBMX-stimulated melanoma was significantly inhibited by DFRB and H, at 106.87 ± 2.32 and 105.63 ± 9.00% of the control (*p* < 0.05). The results illustrated that the anti-melanogenesis effects of DFRB and H are comparable to standard arbutin at the same concentration.

#### 2.4.3. Effects of Sang 5 CMU Extracts on Gene Expression of Melanogenesis-Related Genes

**Figure 6:**
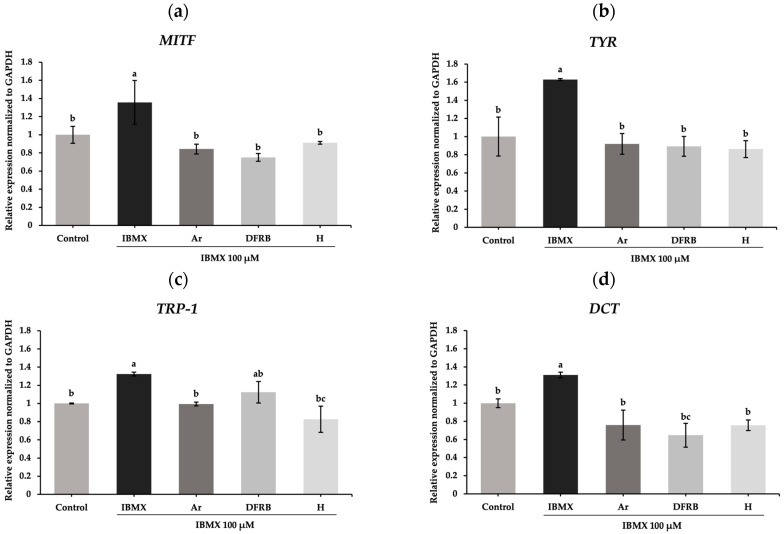
Effects of defatted rice bran (DFRB) and husk (H) extracts of *Oryza sativa* cv. Sang 5 CMU on the gene expression of melanogenesis-related genes (**a**) *MITF*, (**b**) *TYR*, (**c**) *TRP-1*, and (**d**) *DCT* in human melanoma cells after exposure to 100 μM 3-isobutyl-1-methylxanthine (IBMX). Arbutin (Ar) was used as a positive control. Experiments were performed in triplicate. Bars represent mean ± standard deviation. Different superscript letters (a, b, and c) were significantly different at *p* < 0.05, while the same letter showed no significant difference according to a one-way ANOVA test, followed by LSD post hoc test.

To evaluate further, the transcription factor *MITF* and downstream target genes including *TYR*, *TRP-1*, and *DCT* were examined. As demonstrated in Figure 6, IBMX treatment significantly upregulated the genes related to melanogenesis, including *MITF*, *TYR*, *TRP-1*, and *DCT*, with mRNA relative expression of 1.36 ± 0.24, 1.63 ± 0.00, 1.32 ± 0.02, and 1.31 ± 0.03, respectively, compared to untreated cells (*p* < 0.05). Consistent with the results of melanin content and intracellular tyrosinase activity assays, DFRB and H extracts notably inhibited the expression of *MITF*, *TYR*, *TRP-1*, and *DCT* after induction by IBMX (*p* < 0.05). The expression of the *MITF* gene decreased to 0.75 ± 0.04 and 0.91 ± 0.00 after treatment with DFRB and H extracts, respectively (Figure 6a). The gene encoding the tyrosinase enzyme, *TYR*, was also suppressed to 0.89 ± 0.11 and 0.86 ± 0.01 after exposure to DFRB and H, respectively (Figure 6b). As shown in Figure 6c, H extract considerably influenced the mRNA expression of *TRP-1*, with a value of 0.83 ± 0.14. Meanwhile, DFRB showed a strong inhibitory effect on *DCT*, with a level of mRNA expression of 0.65 ± 0.13 (Figure 6d).

### 2.5. Collagen-Synthesis-Promoting Activity

#### 2.5.1. MMP-2 Inhibition Effects of Sang 5 CMU Extracts in Human Fibroblast Cells

**Figure 7:**
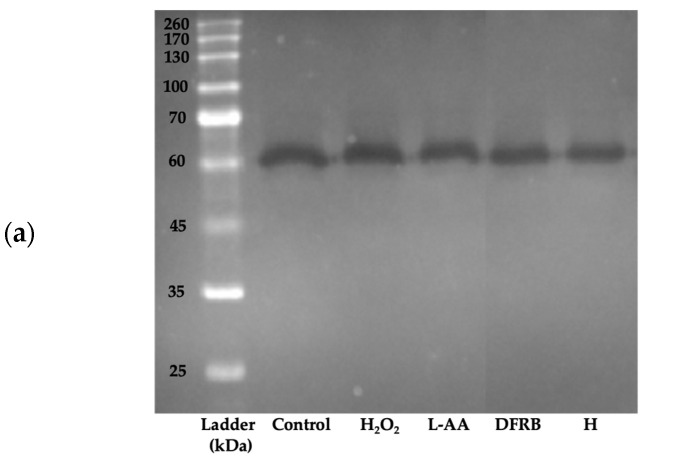

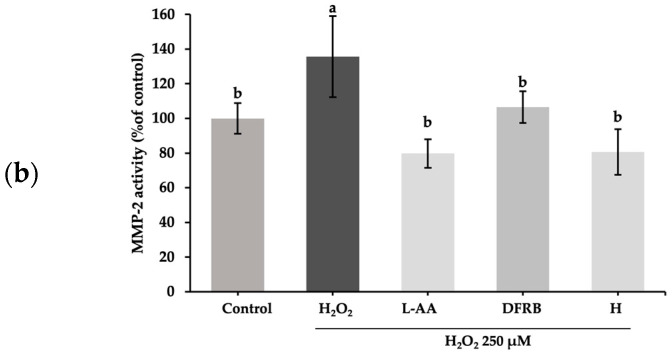
Inhibitory effects of defatted rice bran (DFRB) and husk (H) extracts of *Oryza sativa* cv. Sang 5 CMU on MMP-2 release from fibroblast cells after exposure to 250 μM hydrogen peroxide (H_2_O_2_). (**a**) MMP-2 expression using gelatin zymography and (**b**) MMP-2 activity levels. L-ascorbic acid (L-AA) was used as a positive control. Experiments were performed in triplicate. Bars represent mean ± standard deviation. Different superscript letters (a and b) were significantly different at *p* < 0.05, while the same letter showed no significant difference according to a one-way ANOVA test, followed by LSD post hoc test.

To verify that the reduction in proteolytic enzyme activity was associated with the expression of matrix metalloproteinases in a cell-based model, MMP-2 activity levels were evaluated through gelatin zymography. As demonstrated in Figure 7a, the molecular mass of the active MMP-2 form was approximately 60–65 kDa [22,23]. MMP-2 activity levels were significantly upregulated to 135.71 ± 23.40% of the control after stimulation with H_2_O_2_. After pre-treatment with DFRB and H, the expression of MMP-2 considerably dropped to 106.47 ± 9.12 and 80.67 ± 13.19% of the control, respectively (Figure 7b).

#### 2.5.2. Effects of Sang 5 CMU Extracts on Gene Expression of Collagen-Synthesis-Related Gene

**Figure 8:**
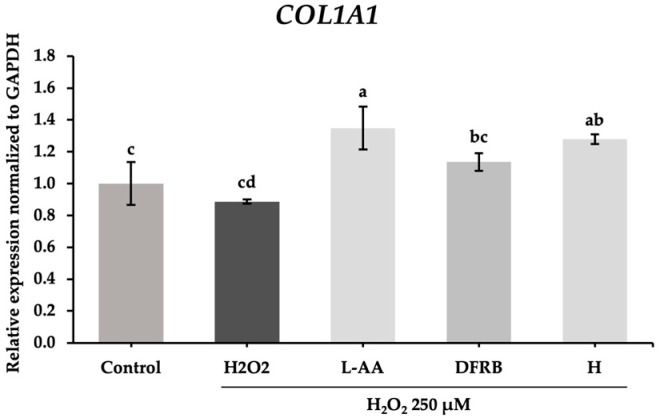
Effects of defatted rice bran (DFRB) and husk (H) extracts of *Oryza sativa* cv. Sang 5 CMU on the gene expression of gene related to the production of collagen, *COL1A1,* in fibroblast cells after exposure to 250 μM hydrogen peroxide (H_2_O_2_). L-ascorbic acid (L-AA) was used as a positive control. Experiments were performed in triplicate. Bars represent mean ± standard deviation. Different superscript letters (a, b, c, and d) were significantly different at *p* < 0.05, while the same letter showed no significant difference according to a one-way ANOVA test, followed by LSD post hoc test.

To confirm whether the inhibitory effect on collagen degradation detected in the prior experiment was related to the alteration of extracellular matrix structure, the expression of genes related to the production of collagen in the fibroblast cells was further investigated. Consistent with the MMP-2 protein expression results, the levels of gene-encoded *COL1A1* were slightly increased to 1.14 ± 0.06 in the DRFB treatment group, whereas H treatment considerably upregulated the mRNA relative expression of *COL1A1* to 1.28 ± 0.03, respectively, as demonstrated in Figure 8.

## 3. Discussion

Prolonged exposure to oxidative stress can lead to cellular damage, stimulate skin inflammation, induce melanin production, and disturb skin homeostasis, ultimately causing visible signs of aging, such as wrinkles, hyperpigmented lesions, and thinning of the skin. Plant-derived natural antioxidant compounds have been extensively studied for their potential to address various skin conditions. These antioxidants are effective at attenuating inflamed-skin lesions like atopic dermatitis, protecting the skin from environmental damage induced by oxidative stress, and also reducing the signs of skin aging [4].

Lipid peroxidation is the major consequence of H_2_O_2_-induced cell damage. MDA is a secondary metabolite that is generated from the oxidation of cell membrane lipids [24]. In agreement with that, after inducting fibroblast cells with H_2_O_2_, MDA production levels increased, as demonstrated in Figure 1. In particular, prolonged exposure of the skin to oxidative stress, especially H_2_O_2_, leads to the depletion of NRF2 via the phosphorylation of glycogen synthase kinase 3β (GSK3β) and a consequent reduction in levels of detoxifying enzymes such as HO-1 and NQO-1 [4]. In this study, the results indicated that treatment with DFRB and H extracts could reduce MDA production in H_2_O_2_-stimulated fibroblast cells (Figure 1). This effect is attributed to the influence of DFRB and H extracts on antioxidant capacity via the NRF2 pathway, caused by the upregulation of *NRF2* and *HO-1* gene expression levels (Figure 2). The activation of the NRF2-mediated pathway as a primary defense mechanism against skin damage, coupled with the dramatic increase in the expression of antioxidant enzymes such as *HO-1* as a result of the effects of DFRB and H extracts, indicated their significant role in enhancing antioxidant defenses against oxidative stress and potential skin damage.

Endogenous nitric oxide (NO) produced by skin cells is part of the defense mechanism of the skin against external stimuli. Indeed, NO production in the skin contributes to diverse responses. For instance, NO could stimulate the activity of tyrosinase, thereby promoting melanogenesis in melanocytes [25]. In the current study, we measured nitrite, a stable degradation product from NO oxidation pathways, using the Griess reagent [26]. Our data revealed that DFRB and H extracts alleviated nitrite production in mouse macrophages. Moreover, DFRB was effective in reducing nitrite accumulation in fibroblast cells following LPS induction (Figure 3).

Alterations of skin homeostasis due to external stimuli can also surge pro-inflammatory cytokines such as IL-1β and IL-6, resulting in skin inflammation [27,28]. It was reported that in mice lacking NRF2, the degree of inflammation is more difficult to return to basal levels compared to wild-type mice [4]. In accordance with this, activation of the NRF2-mediated pathway by DFRB and H extracts reduced the expression of the pro-inflammatory marker *IL-6* after LPS stimulation (Figure 4).

Based on our previous studies, we identified various phenolic and flavonoids compounds in the rice bran and husk parts of Sang 5 CMU. Rice bran contains predominantly quercetin (1.27 ± 0.01 mg/g sample), followed by chlorogenic acid, naringin, epigallocatechin gallate, and epicatechin (0.79 ± 0.01, 0.58 ± 0.08, 0.42 ± 0.03, and 0.22 ± 0.05 mg/g sample, respectively) [19]. On the other hand, rice husk mainly contains naringin, epigallocatechin gallate, and chlorogenic acid (6.60 ± 2.15, 2.80 ± 0.04, and 1.90 ± 0.10 mg/g sample, respectively) [18]. It has been reported that chlorogenic acid, a phenolic compound, exhibits potent inhibition of oxidative damage and inflammatory responses [29,30,31] and prevents skin photoaging [2]. Similarly, naringin, a natural flavanone glycoside, also markedly suppresses oxidative stress and the release of inflammatory cytokines [32,33,34].

A study on quercetin and Kelch-like ECH-associated protein 1 (Keap1)–NRF2 complexes uncovered the underlying mechanisms that drive synergistic effects and enhance antioxidant activity. Hydrogen bonds between quercetin derivatives interact with Arg483, Arg380, Leu365, and Ser508 of the Keap1 domain and contribute to the activating NRF2 function [35]. Furthermore, the residues of targeted inflammatory cytokines such human IL-6, including Glu93 and Glu172, interact with naringin by forming hydrogen bonds [36].

Melanin pigments function as a skin-protective response to environmental damage caused by oxidative stress and inflammation. However, excessive melanin production, coupled with the activation of signaling pathways associated with melanogenesis, not only contributes to signs of skin aging but also plays a role in the progression or development of cutaneous melanoma [37].

Generally, in the synthesis of melanin, the initial substrate, L-tyrosine, is converted by tyrosinase into L-DOPA and L-dopaquinone. The tyrosinase enzyme-mediated first stage is the rate-limiting step in the melanogenesis process. Subsequently, L-dopaquinone is further oxidized to L-dopachrome, eventually leading to the production of melanin pigments [38]. Within the screening of tyrosinase activity, we conducted the mushroom tyrosinase inhibition assay. Our observations revealed that DFRB and H extracts inhibited L-dopachrome formation by disrupting the coupling between the substrate, L-tyrosine, and mushroom tyrosinase (Table 3). Subsequently, we further performed IBMX-induced melanin production in human melanoma cells. IBMX is known to upregulate melanin production through the cAMP cascade [39]. Elevated cAMP levels induce protein kinase A (PKA) translocation into the nucleus, resulting in the activation of *MITF* and the expression of pigmentary genes such as *TYR*, *TRP-1*, and *DCT* [8]. In this study, we found that both extracts suppressed melanin production and intracellular tyrosinase activity in IBMX-induced melanoma cells, as shown in Figure 5. Both extracts lowered the levels of genes related to melanogenesis, including *MITF, TYR*, *TRP-1*, and *DCT,* as illustrated in Figure 6. Coherent with this, epicatechin, found in rice bran of Sang 5 CMU, and epigallocatechin gallate, flavanol molecules that are present in both rice bran and husk, have been reported to have anti-melanogenic effects in B16F10 murine melanoma cells [40,41].

In addition, the correlation analysis revealed correlations between the antioxidant capacity of samples and the development of melanogenesis in melanoma cells, as illustrated in Figures S1 and S2. *NRF2* mRNA expression in fibroblast cells exhibited obvious negative correlations with melanin production, intracellular tyrosinase activity, and the expression of genes related to melanogenesis after DFRB and H treatment. Conversely, the nitrite contents produced by fibroblasts correlated positively with melanin production and intracellular tyrosinase activity in melanoma cells. Accordingly, potent natural antioxidants and anti-inflammatory agents corresponded to lower melanin pigmentation.

The reduction of collagen within the ECM leads to the deterioration of skin structure, contributing to the appearance of wrinkles and thinning of the skin, which are common signs of aging skin. The primary cause of ECM degradation is the activation of MMPs, which are exacerbated by exposure to environmental factors such as radiation, air pollution, or infections. The breakdown of collagen by MMPs elevates oxidative levels within damaged fibroblast cells [9,10]. Consistent with this, our findings regarding high TBARS levels in damaged fibroblasts (Figure 1) corresponded to the results of MMP-2 activity analysis in H_2_O_2_-induced fibroblast cells (Figure 7). The results indicated that oxidative levels, as measured by the TBARS assay, and MMP-2 activity were both elevated in damaged fibroblasts.

Collagen functions to support the dermal ECM and maintain skin strength and integrity. Type I collagen (COL1), encoded by the *COL1A1* and *COL1A2* genes, is the most abundant collagen within the ECM [42]. Additionally, a previous study demonstrated that gene expressions of *COL1A1*, *COL4A1*, and *COL7A1* decreased in human dermal fibroblasts isolated from elderly female donors [43]. Our findings suggest that the effects of DFRB and H extracts on upregulating *COL1A1* gene expression are consistent with their inhibitory activities on MMP-2 (Figure 7 and Figure 8). The relationship between *COL1A1* gene expression and MMP-2 activity in DFRB- and H-treated fibroblasts was evaluated using Pearson correlation. As shown in Figures S1 and S2, we found that the expression of the *COL1A1* gene was negatively correlated with MMP-2 protein expression (Pearson r = −0.883 and −0.635 for DFRB and H treatments, respectively).

This is supported by the fact that quercetin, a flavonol compound found in rice bran and husk parts of Sang 5 CMU, has been reported to prevent collagen degradation and accelerate the healing of skin wounds [44,45,46]. Moreover, epigallocatechin gallate and its derivatives have been shown to protect against collagen degradation and improve collagen fibers in photodamaged skin in mice [47,48]. Furthermore, chlorogenic acid, a type of phenolic acid, has been proven to prevent photoaging by increasing collagen type 1 mRNA and protein expression [2].

Moreover, molecular interactions are crucial for the high biological activity observed in complex systems. A previous study revealed that the linear structure of collagen type I is maintained by a hydrogen bond network within the triple helix structure. These findings illustrated the complex nature of epigallocatechin gallate and collagen interactions, where both non-covalent and covalent bonds contribute to the stability and effectiveness of the complex. These interactions include covalent bonds between C3 and C4 in ring B and Lys935; hydrogen bonds between hydroxyl groups and Ala945, Ala932, and Lys935 in collagen; as well as hydrophobic interactions occurring between the hydrophobic rings of epigallocatechin gallate and Ala945, Ala932, Ala931, Thr946, and Leu934 in collagen [49].

Ethanolic extracts derived from rice bran and husk parts, enriched with bioactive compounds, could be utilized for skin protection. Considering these findings, H extract from Sang 5 CMU, exhibiting superior antioxidant, anti-inflammatory, anti-melanogenic, and collagen-preserving properties compared to DFRB, shows more potential for preventing skin damage. However, further evaluation is necessary to determine their potential based on clinical trials.

## 4. Materials and Methods

### 4.1. Sample Preparation

The rice variety Sang 5 CMU was obtained from the Lanna Rice Research Center, Chiang Mai University, Chiang Mai, Thailand, in October 2021. Herbarium voucher specimens of *O. sativa* var. Sang 5 CMU (PNPRDU65006) were deposited in the Pharmaceutical and Natural Products Research and Development Unit (PNPRDU), Chiang Mai University, Chiang Mai, Thailand. Crude extracts of rice bran and husk extracts were prepared as previously described [13]. In brief, 100 g of rice husks were initially collected and subjected to maceration with 6 L of 95% ethanol for 48 h. Subsequently, de-oiled rice bran (100 g), which was completely stripped of oil using screw pressing and dichloromethane, was soaked in 95% ethanol (6 L) for 48 h. The resulting ethanolic extracts were evaporated at 50 °C using a rotary evaporator (Hei-VAP value, Heidolph, Schwabach, Germany) until completely dried. Ethanolic extracts of defatted rice bran and husk portions of *O. sativa* var. Sang 5 CMU were labeled as DFRB and H, respectively. All samples were stored at 4 °C before further analysis.

### 4.2. Cell Culture and Cell Viability Assessment

Human fibroblast cells (JCRB1006.4F) and G-361 human melanoma (IFO50009) cells were obtained from the JCRB cell bank (Osaka, Japan). Murine RAW 264.7 macrophage cells were obtained from the American Type Culture Collection (Rockville, MD, USA). Human fibroblast and murine macrophage cells were cultured in Dulbecco’s modified Eagle’s medium (Gibco, Grand Island, NY, USA, 121000-046, lot no. 2641717) supplemented with 10% fetal bovine serum and maintained in a humidified incubator at 37 °C with 5% CO_2_. Human melanoma cells were grown in Eagle’s minimal essential medium (Gibco, Grand Island, NY, USA, 41500-034, lot no. 2787087) supplemented with 10% fetal bovine serum (HyClone™ Cytiva, Pasching, Austria, cat no. SV30160.03, lot no. RJ20230003) at 37 °C with 5% CO_2_. All cells from passages 3 to 10 were used in the tests. Prior to sample treatment, cell viability was assessed using the sulforhodamine B (SRB) cytotoxicity assay according to the previous study [14]. Rice bran and husk extracts were diluted within a concentration range of 0.0156 to 1 mg/mL. Cells were seeded into 96-well microplates at the concentration of 1 × 10^3^ cells/well and grown to reach 80% confluency. After that, cells were exposed to each sample for 24 h and 48 h of incubation, respectively. Surviving cells were fixed with 50% trichloroacetic acid solution (PanReac AppliChem, Barcelona, Spain) and stained with 0.04% SRB dye solution (Sigma Aldrich, St. Louis, MO, USA). Plates were washed with 1% acetic acid and left to dry overnight. A 10 mM Tris base (Vivantis, Shah Alam, Selangor, Malaysia) was added to solubilize the dye in living adherent cells. The absorbance of bound dye was measured using a microplate reader at 515 nm. Culture medium was used as the control. Cell viability was calculated from absorbance values as relative percentages of the control group.

### 4.3. Scavenging Activity

#### 4.3.1. DPPH Radical Scavenging Assay

The antioxidant activity against 1,1-diphenyl-2-picrylhydrazyl (DPPH), 2,2′-azino-bis (3-ethylbenzothiazoline-6-sulphonic acid) (ABTS) radicals and ferrous ion–ferrozine complex was determined according to the previous study [13]. Briefly, a sample (100 µL) in range of 0.01–1 mg/mL was reacted with 0.1 mM DPPH solution (50 µL) (Sigma Chemical, St. Louis, MO, USA) for 30 min. The absorbance of the solution was measured at 515 nm. Trolox was used as the positive control. The percentage of DPPH inhibition was calculated from the following formula: the scavenging activity against DPPH radical (%) = [(A − B) − (C − D)]/(A − B) × 100, where A and B represent the absorbance of solvent control with or without DPPH radical, and C and D represent the absorbance of sample with or without DPPH radical, respectively. The scavenging activity against DPPH radicals was expressed as IC_50_ values, which is the sample concentration that yielded 50% DPPH radical scavenging activity.

#### 4.3.2. ABTS Radical Scavenging Assay

First, the ABTS stock solution was prepared by mixing 7 mM ABTS (Sigma Chemical, St. Louis, MO, USA) in 2.45 mM potassium persulfate solution (a ratio of 2:1 *v*/*v*) for 16–18 h. Subsequently, the ABTS stock solution was diluted with water until the absorbance of the ABTS working solution reached 0.7–0.9 units at 734 nm. Each sample (25 µL) was reacted with the ABTS working solution (200 µL) for 10 min. The absorbance of the solution was measured at 734 nm. Trolox was used as the positive control. The percentage of ABTS inhibition was calculated from the following formula: the scavenging activity against ABTS radical (%) = [(A − B) − (C − D)]/(A − B) × 100, where A and B represent the absorbance of solvent control with or without ABTS radical, and C and D represent the absorbance of sample with or without ABTS radical, respectively. The scavenging activity against ABTS radical was expressed as IC_50_ values, which is the sample concentration that yielded 50% ABTS radical scavenging activity.

#### 4.3.3. Ferrous Ion Chelating Assay

Briefly, a sample (100 µL) was mixed with 5 mM ferrozine (50 µL) (Sigma Chemical, St. Louis, MO, USA) and then reacted with 2 mM iron (II) chloride tetrahydrate (FeCl_2_ · 4H_2_O) (Sigma Chemical, St. Louis, MO, USA). The reaction was left at room temperature for 30 min. The absorbance of the solution was measured at 562 nm. EDTA was used as the positive control. The percentage of iron chelation was calculated from the following formula: the chelating activity against iron (%) = [(A − B) − (C − D)]/(A − B) × 100, where A and B represent the absorbance of solvent control with or without ferrous-ferrozine complex, and C and D represent the absorbance of the mixture of sample and ferrous ion with or without ferrozine, respectively. The chelating activity against the ferrous–ferrozine complex was expressed as IC_50_ values, which is the sample concentration that yielded 50% iron chelating activity.

#### 4.3.4. Thiobarbituric Acid Reactive Substances Assay in Human Fibroblast Cells

The levels of reactive metabolites from lipid peroxidation were assessed as previously described [13] by measuring malondialdehyde (MDA) production. Fibroblast cells (2.5 × 10^5^ cells/well) were seeded into 6-well plates. Then, cells were pre-exposed to each sample for 24 h and subsequently incubated with 0.2 mM hydrogen peroxide (H_2_O_2_) for another 24 h. After treatment, cell lysates were collected to react with 0.6% (*w*/*v*) thiobarbituric acid (TBA, BDH Chem. Ltd., Poole, UK) at 100 °C for 10 min. The reaction was stopped immediately by placing it in a freezer (−20 °C) for 5 min. The absorbance of the reaction mixture between TBA and MDA released from cells or thiobarbituric acid reactive substances (TBARS) was measured at 532 nm. L-ascorbic acid (L-AA) was used as the positive control. The level of reactive substances was calculated from absorbance values as relative percentages of the untreated group.

### 4.4. Anti-Inflammatory Activity

#### Nitrite Concentration Assay in Murine Macrophage and Human Fibroblast Cells

The measurement of nitrite concentration was adapted from the previous work [50]. RAW 264.7 macrophage and human fibroblast cells were seeded into 96-well microplates at a concentration of 1 × 10^3^ cells/well and left for 24 h. Cells were pre-treated with each sample for 1 h and further incubated with 1 μg/mL lipopolysaccharides (LPS, Sigma Chemical, St. Louis, MO, USA) for an additional 24 h. The amount of nitrite concentration released in the cell supernatant was evaluated using the Griess reagent kit (Invitrogen, Thermo Fisher Scientific, Eugene, OR, USA, cat no. G7921) according to the manufacturer’s procedure. Diclofenac sodium (DFN) was used as the positive control. The level of nitrite concentration was calculated from a sodium nitrite standard curve in the range of 1–100 μM (y = 0.011x, R^2^ = 0.999).

### 4.5. Anti-Melanogenesis Activity

#### 4.5.1. Mushroom Tyrosinase Inhibition Assay

The inhibitory activity against the mushroom tyrosinase enzyme was performed as previously described [13], with a slight modification. Briefly, each sample (40 µL) was mixed with 0.1 M phosphate buffer (80 µL) and 100 units/mL mushroom tyrosinase (Sigma Chemical, St. Louis, MO, USA) in phosphate buffer (40 µL). Afterward, the mixture solution was reacted with the substrate solution (40 µL), 1.5 mM L-tyrosine (Bio Basic, Markham, ON, Canada). Plates were left for 30 min. After incubation, the absorbance of L-dopachrome formation was measured at 475 nm. Arbutin (Ar) was used as the positive control. The percentage of mushroom tyrosinase inhibition was calculated from the following formula: the inhibition activity against mushroom tyrosinase (%) = [(A − B) − (C − D)]/(A − B) × 100, where A and B represent the absorbance of solvent control with or without mushroom tyrosinase, and C and D represent the absorbance of sample with or without mushroom tyrosinase, respectively. The inhibition activity against mushroom tyrosinase was expressed as IC_50_ values, which is the sample concentration that yielded 50% mushroom tyrosinase inhibition.

#### 4.5.2. Melanin Content Assay in Human Melanoma Cells

Cellular melanin content was measured as previously described [13], with slight modifications. Briefly, melanoma cells (2.5 × 10^5^ cells/well) were seeded into 6-well plates and left for 24 h. Then, cells were pre-treated with samples for 1 h and then exposed to 100 μM 3-isobutyl-1-methylxanthine (IBMX, Sigma Aldrich, St. Louis, MO, USA, cat no. I5879) for another 48 h. Afterward, cells were washed with phosphate-buffered saline and harvested by trypsinization using 0.25% trypsin-EDTA solution (Gibco, Grand Island, NY, USA, 15400-054, lot no. 25337762). Cell suspension was centrifuged at 10,000× *g* for 5 min to obtain the cell pellet. A 1N NaOH solution containing 10% (*v*/*v*) DMSO was used to dissolve the intracellular melanin in the cell pellets at 80 °C for 1 h. The absorbance of the solution was measured at 405 nm. Ar was used as the positive control. Melanin content was calculated from absorbance values as relative percentages of the untreated group.

#### 4.5.3. Intracellular Tyrosinase Inhibition Assay in Human Melanoma Cells

The inhibitory activity against the tyrosinase enzyme was performed according to previous report [13], with some modifications. Briefly, melanoma cells were pre-exposed to each sample for 1 h and further induced by 100 μM IBMX for 48 h. Treated cells were harvested by trypsinization, lysed using 1% Triton X-100 solution (VWR Life Science, Solon, OH, USA), and immediately placed in a freezer (−20 °C) for 30 min. Then, the solution was transferred into the 96-well microplates to react with 5 mM L-dihydroxyphenylalanine (L-DOPA, Sigma Chemical, St. Louis, MO, USA) at 37 °C for 1 h. The absorbance of the reaction mixture was measured at 492 nm. The cellular active tyrosinase level was calculated from absorbance values as relative percentages of the untreated group.

### 4.6. Collagen-Synthesis-Promoting Activity

#### MMP-2 Inhibition Assay in Human Fibroblast Cells

The inhibitory activity against the expression of MMP-2 protein was performed using the gelatin zymography technique, as previously reported [51], with some modifications. Briefly, cell supernatant (10 μL) was loaded onto 10% sodium dodecyl sulfate (SDS-PAGE) gels containing 0.1% gelatin and separated using a gel electrophoresis system (BioRad, Hercules, CA, USA) at 130 V for 80 min. Then, the gels were washed with 2.5% Triton X-100 solution at 37 °C for 1 h and then soaked with buffer containing 50 mM Tris, 5 mM CaCl_2_, and 0.01% sodium azide at 37 °C for 18 h. Gels were stained with 0.5% Coomassie brilliant blue R-250 solution (Bio Basic, Markham, ON, Canada) to visualize the bands. Protein bands were detected using the Gel Doc™ EZ System (Version 3.0; Bio-Rad) and analyzed through the Image Lab™ software 6.1 (Bio-Rad, Hercules, CA, USA). L-AA was used as the positive control. The level of MMP-2 protein was calculated from the band intensity as relative percentages of the untreated group.

### 4.7. Semi-Quantitative Reverse Transcription and Polymerase Chain Reaction

The expression of antioxidant-related genes (*NRF2* and *HO-1*), inflammatory genes (*IL-1β* and *IL-6*), melanogenesis-related genes (*MITF*, *TYR*, *TRP-1*, and *DCT*), as well as regulatory gene of collagen synthesis (*COL1A1*) was determined as previously described [14], with minor modifications. The total RNA of treated cells was extracted using the E.Z.N.A.^®^ Total RNA Kit I (Omega Bio-Tek, Norcross, GA, USA) according to the manufacturer’s procedure. The RNA concentration was determined using the NanoDrop™ One^C^ Microvolume UV-Vis Spectrophotometer (Thermo Fisher Scientific, Waltham, MA, USA). The cDNA was synthesized using the MyTaq™ One-Step RT-PCR Kit (Meridian Bioscience™, BIO-65049, lot no. RA387-B110430, Cincinnati, OH, USA). The sequences of target genes are illustrated in Table 4. Nucleic acid was amplified through DW-T960 Gradient PCR Thermal Cycler (Drawell, Shanghai, China). GAPDH was used as an internal control gene for expression normalization. Afterward, RT-PCR products were loaded onto 1% agarose gel stained with ViSafe Red Gel Stain (Vivantis, Shah Alam, Selangor, Malaysia, SD0103, lot no. 6013). The bands were separated in the gels by electrophoresis at 120 V for 60 min in the chamber containing 1X TAE buffer. The measurement of gene expression was carried out using the Gel Doc™ EZ System and the Image Lab™ software.

### 4.8. Statistical Analysis

All data were obtained in triplicate and are presented as mean ± standard deviation. Statistical analysis of data was performed using one-way analysis of variance (ANOVA), followed by the LSD post hoc test (SPSS 23.0 software, Chicago, IL, USA). Significance was considered at *p*-value < 0.05. The correlation coefficients were calculated using Pearson’s correlation coefficient (r).

## 5. Conclusions

This study demonstrated that DFRB and H extracts from *Oryza sativa* variety Sang 5 CMU, which are composed of phenolic (chlorogenic acid) and flavonoid (naringin, epicatechin, epigallocatechin gallate, and quercetin) contents, possess antioxidant properties against TBARS levels, as evidenced by an increase in *NRF2* and *HO-1* mRNA expression. Additionally, they exhibited anti-inflammatory activities by reducing nitric oxide production in mouse macrophages and human fibroblasts, according to their significant suppression of the pro-inflammatory cytokine *IL-6*. Furthermore, the effects of DRFB and H extracts on anti-melanogenic potential were observed through the inhibition of melanin content and intracellular tyrosinase activity in human melanoma cells, achieved by inhibiting the transcription factor *MITF* and the expression of pigmentary genes *TYR*, *TRP-1*, and *DCT*. Moreover, both extracts enhanced collagen production by stimulating *COL1A1* gene expression and decreasing MMP-2 expression. Taken together, ethanolic extracts from the rice cultivar Sang 5 CMU have potential for various cosmetic functions, including anti-inflammation, anti-wrinkle, and whitening applications. The use of ethanol as a GRAS solvent underscores the safety and suitability of these extracts for incorporation into cosmetic formulations, with a recommended concentration of 1–2% extract.

## Figures and Tables

**Table 1:** Phenolic and flavonoid contents in rice bran and husk samples.

Substances (mg/g Sample)	Defatted Rice Bran Extract	Rice Husk Extract
Caffeic acid	0.16 ± 0.01	0.20 ± 0.03
Epicatechin	0.22 ± 0.05	ND
Epigallocatechin gallate	0.42 ± 0.03	2.80 ± 0.04
*p*-Coumaric	0.23 ± 0.04	0.16 ± 0.00
*o*-Coumaric	0.55 ± 0.03	2.49 ± 0.04
Naringin	0.58 ± 0.08	6.60 ± 2.15
Naringenin	ND	0.33 ± 0.00
Quercetin	1.27 ± 0.01	0.59 ± 0.03
Phytic acid	ND	19.42 ± 0.34
Ferulic acid	0.22 ± 0.00	0.76 ± 0.04
Chlorogenic acid	0.79 ± 0.01	1.90 ± 0.10
Kaempferol	0.07 ± 0.01	0.13 ± 0.00
Hydroxybenzoic acid	0.48 ± 0.03	0.53 ± 0.01

Values represent mean ± standard deviation. ND: non detectable.

**Table 2:** IC_50_ values (mg/mL) of Sang 5 CMU Extracts in DPPH, ABTS Scavenging, and Iron Chelating Assays.

Sample	IC_50_ (mg/mL)		
	DPPH	ABTS	Iron Chelation
Defatted rice bran extract (DFRB)	0.98 ± 0.24 ^b^	0.33 ± 0.00 ^a^	0.70 ± 0.05 ^a^
Husk extract (H)	2.97 ± 0.07 ^a^	0.39 ± 0.00 ^a^	0.51 ± 0.01 ^b^
Trolox	0.42 ± 0.00 ^c^	0.06 ± 0.00 ^b^	ND
EDTA	ND	ND	0.03 ± 0.00 ^c^

Experiments were performed in triplicate. Values represent mean ± standard deviation. ND: not determined; different superscript letters (a, b, and c) were significantly different at *p* < 0.05, while the same letter showed no significant difference according to a one-way ANOVA test, followed by LSD post hoc test.

**Table 3:** IC_50_ values (mg/mL) of Sang 5 CMU Extracts against Mushroom Tyrosinase.

Sample	Mushroom Tyrosinase Inhibition
DFRB	0.49 ± 0.00 ^a^
H	0.27 ± 0.02 ^b^
Arbutin	0.24 ± 0.06 ^b^

Experiments were performed in triplicate. Values represent mean ± standard deviation. DFRB: defatted rice bran extract of *Oryza sativa* cv. Sang 5 CMU; H: husk extract of *Oryza sativa* cv. Sang 5 CMU; different superscript letters (a and b) were significantly different at *p* < 0.05, while the same letter showed no significant difference according to a one-way ANOVA test, followed by LSD post hoc test.

**Table 4:** Primer Sequences.

Gene	Forward Sequence (5′ to 3′)	Reverse Sequence (5′ to 3′)	Reference
*NRF2*	AAACCAGTGGATCTGCCAAC	GTTGGCAGATCCACTGGTTT	Nguyen et al. [52]
*HO-1*	AACTTTCAGAAGGGCCAGGT	ACCTGGCCCTTCTGAAAGTT	Gao et al. [53]
*IL-1β*	CTGAGCTCGCCAGTGAATG	CATTCACTGGCGAGCTCAG	Garcin et al. [54]
*IL-6*	ACTCACCTCTTCAGAACGAATTG	CAATTCGTTCTGAAGAGGTGAGT	Cui et al. [55]
*MITF*	ACCGTCTCTCACTGGATTGGT	ACCAATCCAGTGAGAGACGGT	Javelaud et al. [56]
*TYR*	TTGGCATAGACTCTTCTTGTTGCGG	CCGCAACAAGAAGAGTCTATGCCAA	Javelaud et al. [56]
*TRP-1*	TGGCAAAGCGCACAACTCACCC	GGGTGAGTTGTGCGCTTTGCCA	Javelaud et al. [56]
*DCT*	TGTGGAGACTGCAAGTTTGGC	GCCAAACTTGCAGTCTCCACA	Javelaud et al. [56]
*COL1A1*	GTGCGATGACGTGATCTGTGA	TCACAGATCACGTCATCGCAC	Zhang et al. [57]
*GAPDH*	GGAAGGTGAAGGTCGGAGTC	CTCAGCCTTGACGGTGCCATG	Khantham et al. [14]

## Data Availability

Data are contained within the article and Supplementary Materials.

## Supplementary Materials

The following supporting information can be downloaded at: https://www.mdpi.com/article/10.3390/plants13131795/s1, Figure S1: Pearson’s correlation analysis between biological activities of defatted rice bran (DFRB) extract of *Oryza sativa* cv. Sang 5 CMU; Figure S2: Pearson’s correlation analysis between biological activities of rice husk (H) extract of *Oryza sativa* cv. Sang 5 CMU.
