# Supplementary material for: Antioxidant, Anti-Inflammation, and Melanogenesis Inhibition of Sang 5 CMU Rice (Oryza sativa) Byproduct for Cosmetic Applications
Source: Plants (Basel). 2024 Jun 28;13(13):1795. doi: 10.3390/plants13131795 (PMC11244455; doi:10.3390/plants13131795)
Supplement: Supplementary file 1 [file plants-13-01795-s001.zip › plants-3023055-supplementary.pdf]

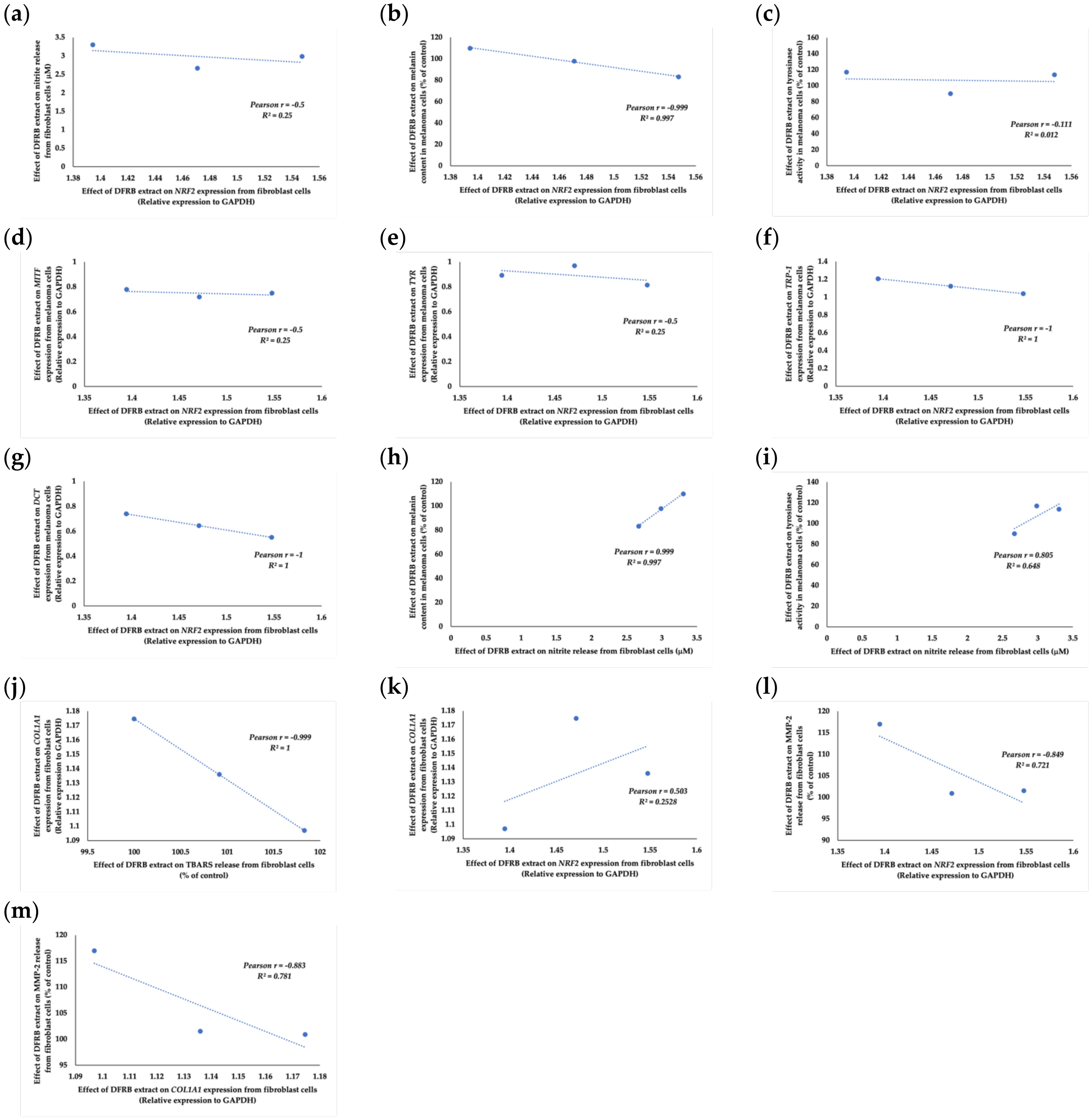

Figure S1. Pearson's correlation analysis between biological activities of defatted rice bran (DFRB) extract of *Oryza sativa* cv. Sang 5 CMU on (**a**) *NRF2* expression and nitrite production in fibroblast cells; (**b**) *NRF2* expression in fibroblast cells and melanin content in melanoma cells; (**c**) *NRF2* expression in fibroblast cells and tyrosinase activity in melanoma cells; (**d**) *NRF2* expression in fibroblast cells and *MITF* expression in melanoma cells; (**e**) *NRF2* expression in fibroblast cells and *TYR* expression in melanoma cells; (**f**) *NRF2* expression in fibroblast cells and *TRP-1* expression in melanoma cells; (**g**) *NRF2* expression in fibroblast cells and *DCT* expression in melanoma cells; (**h**) nitrite production in fibroblast cells and melanin content in melanoma cells; (**i**) nitrite production in fibroblast cells and tyrosinase activity in melanoma cells; (**j**) *COL1A1* expression and TBARS production in fibroblast cells; (**k**) *COL1A1* expression and *NRF2* expression in fibroblast cells; (**l**) *NRF2* expression and MMP-2 activity in fibroblast cells; (**m**) *COL1A1* expression and MMP-2 activity in fibroblast cells.

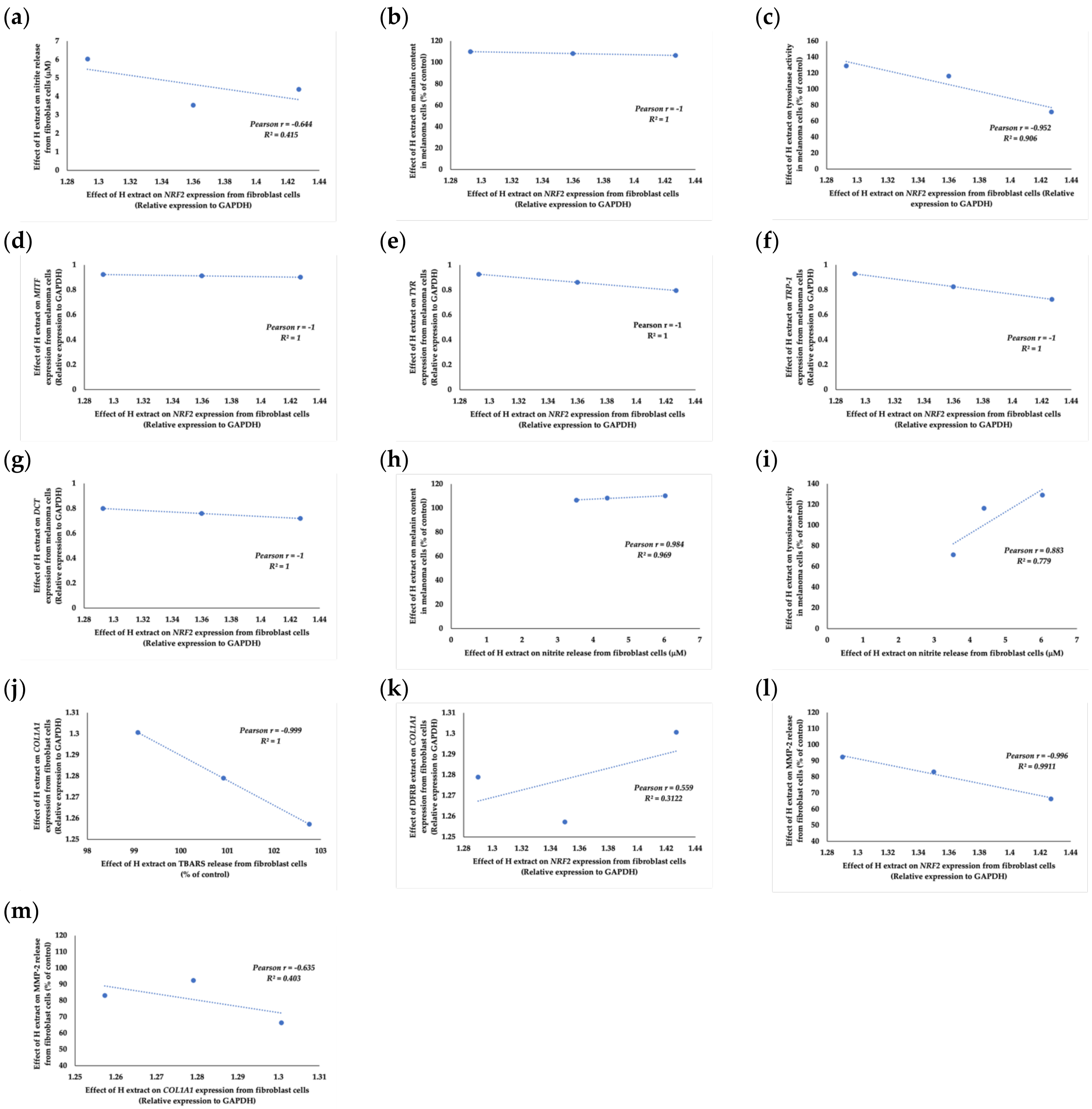

Figure S2. Pearson's correlation analysis between biological activities of rice husk (H) extract of *Oryza sativa* cv. Sang 5 CMU on (**a**) *NRF2* expression and nitrite production in fibroblast cells; (**b**) *NRF2* expression in fibroblast cells and melanin content in melanoma cells; (**c**) *NRF2* expression in fibroblast cells and tyrosinase activity in melanoma cells; (**d**) *NRF2* expression in fibroblast cells and *MITF* expression in melanoma cells; (**e**) *NRF2* expression in fibroblast cells and *TYR* expression in melanoma cells; (**f**) *NRF2* expression in fibroblast cells and *TRP-1* expression in melanoma cells; (**g**) *NRF2* expression in fibroblast cells and *DCT* expression in melanoma cells; (**h**) nitrite production in fibroblast cells and melanin content in melanoma cells; (**i**) nitrite production in fibroblast cells and tyrosinase activity in melanoma cells; (**j**) *COL1A1* expression and TBARS production in fibroblast cells; (**k**) *COL1A1* expression and *NRF2* expression in fibroblast cells; (**l**) *NRF2* expression and MMP-2 activity in fibroblast cells; (**m**) *COL1A1* expression and MMP-2 activity in fibroblast cells.
